# Evaluation of the importance of fever with respect to dengue prognosis according to the 2009 WHO classification: a retrospective study

**DOI:** 10.1186/s12879-016-2128-4

**Published:** 2017-01-04

**Authors:** Caroline Tukasan, Nathália Barbosa Furlan, Cássia Fernanda Estofolete, Maurício Lacerda Nogueira, Natal Santos da Silva

**Affiliations:** 1 Faculdade de Medicina, União das Faculdades dos Grandes Lagos, São José do Rio Preto, São Paulo Brazil; 2 Laboratório de Pesquisas em Virologia, Faculdade de Medicina de São José do Rio Preto, São José do Rio Preto, São Paulo Brazil; 3 Laboratório de Modelagens Matemática e Estatística em Medicina, Faculdade de Medicina, União das Faculdades dos Grandes Lagos, São José do Rio Preto, São Paulo Brazil

**Keywords:** Dengue fever, Severe dengue, Dengue epidemiology, WHO 2009 classification

## Abstract

**Background:**

The 2009 revised World Health Organization (WHO) guidelines for dengue describe fever as the core symptom. Accordingly, the diagnosis of non-febrile patients is complicated. The aim of this study was to evaluate the importance of fever in patients with dengue according to the 2009 revised WHO classification.

**Methods:**

In this study, we assessed 30,670 dengue cases using enzyme-linked immunosorbent assay, detection of the non-structural protein 1, or polymerase chain reaction for diagnostic confirmation. Fisher’s exact test was used to evaluate associations between fever and related clinical manifestations. The Mann–Whitney *U* test was used to assess the association of dengue classification with fever and time to treatment. The effects of fever and time to treatment on the risk of progression were analyzed using an ordinal logistic regression to stereotype the model.

**Results:**

Disease classification was found to associate significantly with both fever and time to treatment (both *P* < 0.001). Non-febrile patients were nearly four-fold more likely to exhibit “dengue without warning signs” than “severe dengue” (odds ratio [OR] = 3.74; 95% confidence interval [CI]: 3.20–4.36). Patients who received treatment within 7 days were twice as likely to have “dengue without warning signs” as opposed to “severe dengue” when compared to those who waited >7 days (OR = 2.23; 95% CI: 1.78–2.80). However, this difference was negligible in the multivariate analysis (OR = 1.02; 95% CI: 0.98–1.07).

**Conclusions:**

Fever is a risk factor for disease progression in patients with dengue. However, non-febrile patients should not be neglected because this may delay treatment and could lead to more severe disease.

**Electronic supplementary material:**

The online version of this article (doi:10.1186/s12879-016-2128-4) contains supplementary material, which is available to authorized users.

## Background

Dengue is an infectious disease that exhibits acute evolution [1]. The incidence of dengue has increased across all age groups [2] to become a clear public health problem [3]. The causative dengue virus (DENV) belongs to the *Flaviviridae* family within the *Flavivirus* genus [4], and harbors RNA genetic material that encodes three structural (capsid [C], membrane protein [M] and glycoprotein for the viral envelope [E]) and seven non-structural (NS1, NS2a, NS2b, NS3, NS4a, NS4b and NS5) proteins [5] essential for viral replication in host cells [4, 6]. DENV is transmitted by a bite from a female *Aedes aegypti* mosquito [7] that caries one of the four virus serotypes [4]. The viremic period of the disease is defined as the period during which dengue virus can be detected in the blood of an infected human [6]. Following inoculation of the virus into the host, the incubation period may vary from 4 to 10 days [8, 9], after which various clinical signs and symptoms will emerge [9]. Infection with a specific virus serotype initiates permanent, or homotypic, immunity against that serotype [5, 6, 9]. Reinfection with a different strain may induce a cross-reaction in which existing antibodies from the first infection bind but do not abolish the virus; this process generally leads to more severe disease [5, 10].

The 1997 World Health Organization (WHO) guidelines on dengue presented three disease categories: dengue fever, dengue hemorrhagic fever, and dengue shock syndrome [3]. Investigations intended to classify the disease and facilitate the initiation of treatment are necessary when a patient is suspected to have dengue. Patients with dengue fever usually presents with an abrupt-onset high fever [11] and two or more other symptoms, such as headache, retro-orbital pain, myalgia, and arthralgia. Laboratory tests may indicate leukopenia and thrombocytopenia without plasma leakage. In addition to the classic symptoms of dengue fever, patients with dengue hemorrhagic fever present with a positive result on the tourniquet test and signs of spontaneous bleeding. This disease is divided into four grades, of which grades three and four are defined as dengue shock syndrome, indicated by the presence of signs of circulatory deficiency such as a weak pulse and hypotension, or even an absent pulse and blood pressure, as a result of plasma leakage [8].

Owing to difficulties with the application of this classification to patients with more severe symptoms, the WHO revised its guidelines in 2009 to include three categories: dengue without warning signs, dengue with warning signs, and severe dengue [2, 3, 12]. Symptoms of dengue with warning signs may include abdominal pain, vomiting, fluid retention, bleeding from mucous membranes, lethargy, and hepatomegaly. Laboratory tests may indicate an increase in erythrocytes with thrombocytopenia. Patients with severe dengue experience plasma leakage, bleeding, and organ failure [2, 3, 8]. Notably, fever is the core symptom of both classifications. Patients who do not present with fever or who develop fever later are difficult to classify according to these guidelines. Furthermore, dengue may present with symptoms similar to other acute febrile illnesses, especially those caused by other arboviruses such as the Zika and Chikungunya viruses, which were both recently detected in Brazil (in 2015 and 2014, respectively), thus contributing to the complicated diagnosis of dengue [13].

The main objective of the present study was to evaluate the importance of fever in patients with dengue, to analyze clinical data related to fever, and to assess associations between fever and the severity indices listed in the 2009 revised WHO guidelines.

## Methods

This study evaluated 30,670 confirmed cases of dengue identified in São José do Rio Preto, São Paulo, Brazil between April 1998 and June 2012 (before the emergence of the Zika and Chikungunya viruses in Brazil). Patient information was collected from medical forms of the dengue surveillance system (SINAN, Information System for Notifiable Diseases) associated with the Brazilian Ministry of Health (Additional file 1). This service requires notification of all suspected cases of this disease nationwide. Diagnostic confirmation was conducted in government laboratories via the enzyme-linked immunosorbent assay (ELISA)-based detection of specific IgM antibodies, detection of the non-structural protein 1 (NS1), or polymerase chain reaction (PCR).

The classification system proposed in the WHO 2009 guidelines was used as an ordinal dependent variable. The outcome was one of three different classification levels: dengue without warning signs, dengue with warning signs, and severe dengue. The presence of fever reported by the patient or measured at the time of medical examination was evaluated separately as an independent variable. The time to treatment from the onset of any dengue symptom was categorized as ≤7 days or >7 days and also analyzed as an independent variable. General clinical manifestations, such as headache, vomiting, arthralgia, myalgia, prostration, and diarrhea, were grouped as a single variable (general symptoms). Hemorrhagic manifestations were also included as an independent variable and included the clinical presentation of epistaxis, petechiae, rash, gingival bleeding, hematuria, gastrointestinal bleeding, menorrhagia, and positive tourniquet test. Ascites, pleural effusion, pericardial effusion, hematocrit elevation above the reference levels for sex and age, and the presence of hypoproteinemia were grouped as a plasma leakage variable. Organ failure, including myocarditis, neurological manifestations (as impaired consciousness), and liver failure (AST or ALT > =1000 IU/mL), as defined by WHO, were considered another variable [8]. All suspected cases of dengue which are notified to the Brazilian Ministry of Health are previously evaluated by a doctor or nurse. In our study we do not consider whether the patient was or was not referred to a hospital for both febrile cases as non-febrile.

Fisher’s exact test was used to assess the associations of fever with sex, general symptoms, hemorrhagic manifestations, plasma leakage, organ failure, and time to treatment. The Mann–Whitney *U* test was used to verify associations of the WHO 2009 classification for dengue with fever and the time to treatment. An ordinal logistic regression to stereotype model was used to analyze the risk of progression to different levels in the WHO 2009 classification among patients with or without fever. Severe dengue was used as the reference category in the pairwise comparison for each classification. The time interval from symptom onset to presentation for medical care was also analyzed. Odds ratios (ORs) were defined as the ratio of the likelihood of dengue without warning signs or severe dengue in the first comparison and of the likelihood of dengue with warning signs or severe dengue in the second comparison. The quality-of-fit of the models was assessed using the deviance test. All statistical tests were performed using SPSS (version 19; SPSS, Inc., Chicago, IL, USA) and the free statistical program R (version 3.2.0, VGAM package rrvglm function; The R Project for Statistical Computing, Vienna, Austria), and a 5% significance level was implemented.

This study was authorized by the Ethical Committee of the União das Faculdades dos Grandes Lagos, São José do Rio Preto, São Paulo, Brazil. The requirement for informed patient consent was waived.

## Results

Of the 30,670 patients with dengue recorded during the study period, fever information was not recorded in the medical forms to 15,914 patients; however, in 14,756 forms this information was present properly. Of these, 14.248 (96.6%) had fever and 508 (3.4%) had not. Only patients with adequate record about the occurrence of were evaluated statistically. 11,726 (79.5%) were aged between 15 and 60 years; 1702 (11.5%) were older than 60 years, and 1328 (9.0%) were aged 0–14 years. The study included 8711 (59.0%) female and 5996 (40.6%) male patients (information on sex was not available for 49 (0.3%) individuals).

Information about general symptoms was available for all 14,756 individuals. Of these, 14,717 (99.7%) exhibited general symptoms, whereas 39 (0.3%) patients reported no general complaints. Of the 14,744 patients with avaliable information about hemorrhagic manifestations, 9320 (63.2%) presented with at least one hemorrhagic manifestation, whereas 5424 (36.8%) had no bleeding complaints patients (information on hemorrhagic manifestation was not available for 12 (0.1%) individuals). At least one form of plasma extravasation was reported in 427 (2.9%) patients, whereas 12,417 (84.1%) had no related complaints (information on plasma extravasation was not available for 1912 (13.0%) individuals). There were 5041 (34.2%) reports of organ dysfunction, whereas 8920 (60.4%) had no dysfunction complaints reported. Regarding the revised WHO classification, 1499 (10.2%), 12,889 (87.3%), and 368 (2.5%) patients were diagnosed with dengue without warning signs, dengue with warning signs, and severe dengue, respectively. The average time to treatment was 5.84 days (standard deviation, 5.25 days).

There was a significantly higher incidence of fever among women (n = 8348, 58.8%) vs. men (n = 5854, 41.2%; *P* < 0.001). Fever was also associated with an increased incidence of general symptoms (*P* < 0.001). In contrast, fever was not statistically significantly associated with hemorrhagic manifestations (*P* = 0.261) or plasma leakage (*P* = 0.689). Despite a significant association of fever with organ failure (*P* = 0.011) 150 patients who did not present with fever were diagnosed with failure of at least one of the studied organs. Fever was not statistically significantly associated with the time to treatment (*P* = 0.133; Table 1).

**Table 1:** Comparison between clinical variables and the presence of fever in patients with dengue

	Gender*		General symptomatology*		Hemorrhagic manifestations**		Plasma leakage^†^		Organ failure^††^		Time to treatment^‡^	
	Male *n* (%)	Female *n* (%)	Yes *n* (%)	No *n* (%)	Yes *n* (%)	No *n* (%)	Yes *n* (%)	No *n* (%)	Yes *n* (%)	No *n* (%)	≤7 days *n* (%)	>7 days *n* (%)
Fever												
Yes	5,854 (41.2)	8,348 (58.8)	13,960 (100.0)	0 (0)	8987 (63.1)	5250 (36.9)	411 (3.3)	11,985 (96.7)	4,891 (36.3)	8,581 (63.7)	11,132 (78.7)	3,008 (21.3)
No	142 (28.1)	363 (71.9)	469 (92.3)	39 (7.7)	333 (65.7)	174 (34.3)	16 (3.6)	432 (96.4)	150 (30.7)	339 (69.3)	380 (75.8)	121 (3.9)

Fisher’s exact test: *P < 0.001, **P = 0.261, ^†^ *P* = 0.689, ^††^ *P* = 0.011, ^‡^ *P* = 0.133

The Mann–Whitney test indicated a statistically significant association between fever and the WHO classification (*U* = 3,160,348.5; *P* < 0.001). A significant association was also observed between the time to treatment and WHO classification (*U* = 43,705,568.5; *P* < 0.001). The ordinal logistic regression was adjusted using the stereotype model, and weights were estimated as model parameters. The univariate analysis indicated that the significant association detected by the Mann–Whitney test represented an increased likelihood that non-febrile individuals would present with “dengue without warning signs” rather than “severe dengue”; this likelihood was nearly four-fold higher than that of febrile individuals (OR = 3.73; 95% confidence interval [CI]: 3 19–4.36). In contrast, the likelihood that patients with or without fever would present with “dengue with warning signs” or “severe dengue” was similar (OR = 1.04; 95% CI: 0.89–1.21).

Patients who presented for treatment within 7 days of symptom onset were twice as likely to be classified as having “dengue without warning signs” rather than “severe dengue”, compared to those who presented for treatment more than 7 days after symptom onset. When these two variables were evaluated in the same multivariate analysis model, the magnitude of the association between patients without fever who presented with “dengue without warning signs” vs. “severe dengue” was retained (OR = 3.74; 95% CI: 3.20–4.36). However, the difference between patients who received treatment within 7 days or >7 days of symptom onset was negligible (OR = 1.02; 95% CI: 0.98–1.07) when “dengue without warning signs” and “severe dengue” were considered. All models exhibited good fit in the deviance test (*P* > 0.05; Table 2).

**Table 2:** Ordinal logistic regression (stereotype model)^a^

	Dengue without warning signs vs. severe dengue			Dengue with warning signs vs. severe dengue		
	β_1_	Odds ratio	95% confidence interval	β_2_	Odds ratio	95% confidence interval
Univariate						
Fever						
Yes^b^		1.00			1.00	
No	1.31	3.73	3.19–4.36	0.04	1.04	0.89–1.21
Time to treatment						
> 7 days^b^		1.00			1.00	
≤ 7 days	0.80	2.23	1.78–2.80	−0.09	0.92	0.73–1.15
Multivariate						
Fever						
Yes^b^		1.00			1.00	
No	1.32	3.74	3.20–4.36	0.04	1.04	0.89–1.22
Time to treatment						
> 7 days^b^		1.00			1.00	
≤ 7 days	0.02	1.02	0.98–1.07	0.0006	1.00	0.96–1.04

Deviance test: *P* > 0.05 for all models

^a^Dependent variable: 2009 World Health Organization guideline dengue classification

^b^Reference

## Discussion

Previous literature reports have not utilized such a large number of patients with both clinically and laboratory confirmed dengue nor a stereotype model to evaluate the importance of fever as a trigger for the investigation of suspected dengue cases. Furthermore, the present study is the first to use the 2009 revised WHO classification system as an outcome measure. Although the results demonstrated a statistically significant association between fever and organ failure (*P* = 0.011), 150 patients without fever also experienced organ failure. Both univariate and multivariate logistic regression analyses indicated that patients without fever were less likely to have severe disease (OR = 3.73; 95% CI: 3.19–4.36 and OR = 3.74; 95% CI: 3.20–4.36, respectively). Furthermore, in the univariate analysis, patients who received treatment within 7 days were less likely to have severe disease when compared to those who received a delayed diagnosis and treatment (OR = 2.23; 95% CI: 1.78–2.80); however, this difference was negligible in the multivariate analysis (OR = 1.02; 95% CI: 0.98–1.07).

The presence of fever, as well as the tourniquet test [11], in suspected dengue carriers is often highly valued as a method of defining the continuity of the conducting case. Even the 2009 WHO guidelines emphasize the presence of fever as a starting point for dengue evaluation [8, 14]. However, the absence of fever, observed in 508 of our study participants, might reduce the demand for medical care. We note that our results highlight the fact that patients without fever were more likely to remain in the lower strata of the 2009 revised classification, and therefore those with fever presented with more severe cases of dengue [8, 11, 14].

Generally, fever has been present in most [15–17], if not all, patients participating in studies of dengue [18]; however, a lack of fever in patients with dengue might be an obstacle to the initiation of investigations and the classification of arbovirosis as dengue without warning signs, dengue with warning signs, or severe dengue. Fever might not be important in the detection of dengue, as shown by Ho et al. in a 2013 study of 581 patients from a hospital in Taiwan in which fever was found to have a moderate sensitivity (67.3%) and positive predictive value (71.7%) and a low specificity (12.3%) and negative predictive value (10.2%) [19]. Reliance on the presentation of fever for a dengue diagnosis might lead to delayed treatment and an increase in disease severity. Furthermore, in a study conducted in the State of Minas Gerais, Brazil, 5.1% of patients with dengue who were admitted to an intensive care unit had not presented with fever at any time prior to admission [20]. Additionally, asymptomatic or presymptomatic individuals might present a high risk of DENV transmission to mosquitos (OR = 5.10; CI: 1.76–57.51 and OR = 4.84; CI: 2:02–11:58, respectively) [21].

Detection of the Chikungunya and Zika viruses in Brazil in 2014 [22] and 2015 [13], respectively, has complicated the diagnosis of dengue based on the presence of fever because infections with these arboviruses yield similar clinical presentations [13]. In addition, cases of Zika might also present clinically as afebrile, as described in a study by Yap [23], leading to the misdiagnosis of dengue as Zika and possibly delaying the initiation of appropriate treatment, leading to further clinical complications. In our cohort of patients with dengue, delays in presentation for treatment were associated with a worse prognosis. Similar results were also found in another study that used a threshold of 4 days with regard to presentation for treatment [19, 24].

We note that we have implemented all precautions to eliminate potential errors that are common in very large retrospective databases, particularly with regard to the annotation of patient records. Additionally, we believe that correct and exhaustive debugging of our data, which involved the removal of conflicting information, increased the reliability of our obtained results and minimized the limitations associated with this type of study.

## Conclusions

We conclude that the presence of fever in patients with dengue can be a risk factor for progression to more severe disease. However, patients who do not present with fever should not be neglected, as this might delay treatment, another factor associated with the development of severe disease.

## Additional file

Additional file 1: SINAM (National Medical Registration of Dengue Cases) Instrument. (PDF 252 kb)

## Abbreviations

CI: Confidence interval
DENV: Dengue virus
ELISA: Enzyme-Linked Immunoassay
NS1: Nonstructural protein 1
NS2a: Nonstructural protein 2a
NS2b: Nonstructural protein 2b
NS3: Nonstructural protein 3
NS4a: Nonstructural protein 4a
NS4b: Nonstructural protein 4b
NS5: Nonstructural protein 5
OR: Odds ratio
PCR: Polymerase chain reaction
SINAN: Notifiable diseases information system
WHO: World Health Organization
