# Supplementary material for: Evaluation of the importance of fever with respect to dengue prognosis according to the 2009 WHO classification: a retrospective study
Source: BMC Infect Dis. 2017 Jan 4;17:6. doi: 10.1186/s12879-016-2128-4 (PMC5209937; doi:10.1186/s12879-016-2128-4)
Supplement: Additional file 1: — SINAM (National Medical Registration of Dengue Cases) Instrument. (PDF 252 kb) [file 12879_2016_2128_MOESM1_ESM.pdf]

MINISTÉRIO DA SAÚDE
SECRETARIA DE VIGILÂNCIA EM SAÚDE
DEPARTAMENTO DE VIGILÂNCIA EPIDEMIOLÓGICA
COORDENAÇÃO GERAL DE DOENÇAS TRANSMISSÍVEIS
GT-SINAN

SISTEMA DE INFORMAÇÃO DE AGRAVO DE NOTIFICAÇÃO - SINAN
DICIONÁRIO DE DADOS – AMBIENTE WINDOWS

AGRAVO: **DENGUE**

CAMPO DA FICHA	BANCO DE DADOS (DBF)					
**NOME**	**TIPO**	**TAM**	**NOME**	**CATEGORIAS**	**DESCRIÇÃO**	**CARACTERISTICAS/ CRÍTICA DE CONSISTENCIA**
Nº de notificação e campos de 1 a 25 dos blocos " Dados Gerais" , " Dados do Caso" e " Dados de residência " correspondem aso mesmos campos da ficha de notificação (ver dicionário de dados da ficha de notificação						
Data de investigação	Data		ANT_DT_INV		Data em que ocorreu a investigação – informar a Data da 1ª visita ao paciente	Data da investigação > ou = Data da notificação<br>Campo obrigatório
Ocupação/ Ramo de Atividade Econômica	Caracter	5	OCUPACAO		Informar a atividade exercida pelo paciente no setor formal, informal ou autônomo ou sua última atividade exercida quando paciente for desempregado. O ramo de atividade econômica do paciente refere-se as atividades econômicas desenvolvidas nos processos de produção do setor primário (agricultura e extrativismo); secundário (indústria) ou terciário (serviços e comércio)	
Deslocamento					Deslocamento (Datas e locais freqüentados no período de 15 meses anteriores ao início dos sinais e sintomas	Preenchimento apenas na ficha
Teve dengue antes	Caracter	1	DENGUE	1- Sim<br>2- Não<br>9- Ignorado	Se o paciente já teve dengue anterior a esta notificação	Se categoria = 2ou = 9, pular para campo 31<br>(vacinado contra febre amarela)
Caso sim, ano	Caracter	4	ANO	Ex: 1999	Ano em que adoeceu de dengue anteriormente	
Vacinado contra febre amarela	Caracter	1	VACINADO	1- Sim<br>2- Não<br>9- Ignorado		Se categoria = 2 ou 9 pular para campo 33 (febre)
Data da última dose	Data		DT_DOSE		Dia, mês e ano da última dose da vacina contra febre amarela	

Sinais e sintomas Febre	Caracter	1	FEBRE	1- Sim<br>2- Não<br>9- Ignorado	Se o paciente relatou febre com duração máxima de 7 dias	Se categoria = 2 ou = 9, pular para campo 36 (prova do laço)
Data do início	Data		DT_FEBRE		Dia, mês e ano do início da febre	
**CAMPO DA FICHA**	**BANCO DE DADOS (DBF)**					
**NOME**	**TIPO**	**TAM**	**NOME**	**CATEGORIAS**	**DESCRIÇÃO**	**CARACTERISTICAS/<br>CRÍTICA DE CONSISTENCIA**
Duração da febre em dias	Numérica	2	DURACAO			
prova do laço	Caracter	1	LACO	1- Positiva<br>2- Negativa<br>3- Não realizada	Resultado da prova do laço	
Outros sinais e sintomas Cefaléia	Caracter	1	CEFALEIA	1- Sim<br>2- Não<br>9- Ignorado	Paciente relatou presença de exantema	
Outros sinais e sintomas dor retro-orbitária	Caracter	1	DOR	1- Sim<br>2- Não<br>9- Ignorado	Paciente relatou dor retroorbitária	
Outros sinais e sintomas mialgia	Caracter	1	MIALGIA	1- Sim<br>2- Não<br>9- Ignorado	Paciente relatou mialgia	
Outros sinais e sintomas Artralgia	Caracter	1	ARTRALGIA	1- Sim<br>2- Não<br>9- Ignorado	Paciente relatou artralgia	
Outros sinais e sintomas Diarréia	Caracter	1	DIARREIA	1- Sim<br>2- Não<br>9- Ignorado	Paciente teve diarréia	
Outros sinais e sintomas Exantema	Caracter	1	EXANTEMA	1- Sim<br>2- Não<br>9- Ignorado	Paciente relatou cefaléia	
Outros sinais e sintomas prostração	Caracter	1	PROSTACAO	1- Sim<br>2- Não<br>9- Ignorado	Paciente relatou prostração	
Outros sinais e sintomas náuseas/vômitos	Caracter	1	NAUSEAS	1- Sim<br>2- Não<br>9- Ignorado	Paciente apresentou náusea	
Outros sinais e sintomas Outros	Caracter	1	OUTROS	1- Sim<br>2- Não<br>9- Ignorado	Se o paciente apresentou outros sinais e sintomas	Se categoria = 1 habilitar para especificar outros
Outros especificar	Caracter	20	SIN_OUT		Descrever quais foram outros sinais e sintomas	

Manifestações hemorrágicas espontâneas Epistaxe	Caracter	1	EPISTAXE	1- Sim<br>2- Não<br>9- Ignorado		
**CAMPO DA FICHA**	**BANCO DE DADOS (DBF)**					
**NOME**	**TIPO**	**TAM**	**NOME**	**CATEGORIAS**	**DESCRIÇÃO**	**CARACTERISTICAS/ CRÍTICA DE CONSISTENCIA**
Manifestações hemorrágicas espontâneas Gengivorragia	Caracter	1	GENGIVO	1- Sim<br>2- Não<br>9- Ignorado		
Manifestações hemorrágicas espontâneas Hematúria	Caracter	1	HEMATURA	1- Sim<br>2- Não<br>9- Ignorado		
Manifestações hemorrágicas espontâneas Petéquias	Caracter	1	PETEQUIAS	1- Sim<br>2- Não<br>9- Ignorado		
Manifestações hemorrágicas espontâneas Metrorragia	Caracter	1	METRO	1- Sim<br>2- Não<br>9- Ignorado		
Manifestações hemorrágicas espontâneas Sangramento Gastrointestinal<br><br>Manifestações hemorrágicas espontâneas Outras	Caracter<br><br>Caracter	1<br><br>1	SANGRAM<br><br>OUTROS_M	1- Sim<br>2- Não<br>9- Ignorado<br><br>1- Sim<br>2- Não<br>9- Ignorado	Paciente apresentou outras manifestações hemorrágicas espontâneas	Se categoria = 1 habilitar para especificar outras
Outras especificar	Caracter	20	OUTROS_M_D		Descrever quais foram outras manifestações hemorrágicas espontâneas	
Derrame cavitário Ascite	Caracter	1	ASCITE	1- Sim<br>2- Não<br>9- Ignorado		
Derrame cavitário Derrame pleural	Caracter	1	PLEURAL	1- Sim<br>2- Não<br>9- Ignorado		
Derrame cavitário Derrame pericárdico	Caracter	1	PERICARDI	1- Sim<br>2- Não<br>9- Ignorado		

Sinais de alerta e/ou gravidade dor abdominal intensa	Caracter	1	ABDOMINAL	1- Sim<br>2- Não<br>9- Ignorado	Paciente relatou dor abdominal intensa	
**CAMPO DA FICHA**	**BANCO DE DADOS (DBF)**					
**NOME**	**TIPO**	**TAM**	**NOME**	**CATEGORIAS**	**DESCRIÇÃO**	**CARACTERISTICAS/ CRÍTICA DE CONSISTENCIA**
Sinais de alerta e/ou gravidade Hipotensão arterial /postural	Caracter	1	HIPOTENSAO	1- Sim<br>2- Não<br>9- Ignorado	Paciente apresenta Hipotensão arterial/postural	
Sinais de alerta e/ou gravidade Manifestações neurológicas	Caracter	1	MANIFESTA	1- Sim<br>2- Não<br>9- Ignorado	Manifestações neurológicas	
Sinais de alerta e/ou gravidade Hepatomegalia dolorosa	Caracter	1	HEPATO	1- Sim<br>2- Não<br>9- Ignorado	Paciente apresenta hepatomegalia dolorosa	
Sinais de alerta e/ou gravidade Choque hipovolêmico	Caracter	1	CHOQUE	1- Sim<br>2- Não<br>9- Ignorado	Paciente apresenta situação de choque hipovolêmico	
Sinais de alerta e/ou gravidade Insuficiência hepática	Caracter	1	INSUFICIEN	1- Sim<br>2- Não<br>9- Ignorado	Paciente apresenta situação de insuficiência hepática	
Sinais de alerta e/ou gravidade Miocardite	Caracter	1	MIOCARDI	1- Sim<br>2- Não<br>9- Ignorado	Paciente apresenta situação de miocardite	
Sinais de alerta e/ou gravidade Outros	Caracter	1	OUTRO_S	1- Sim<br>2- Não<br>9- Ignorado	Paciente apresentou outros sinais de alerta e/ou gravidade	Se categoria = 1 habilitar para especificar outros
Outros especificar	Caracter	20	OUTRO_S_D		Descrever quais foram outros sinais de alerta e/ou gravidade	
Data do Choque ou hipotensão	Data		DT_CHOQUE			
Ocorreu Hospitalização?	Caracter	1	HOSPITALIZ	1- sim<br>2- não<br>9 – ignorado	Se ocorreu hospitalização com o paciente, decorrente de doença exantemática	Se hospitalização = 2 ou 9 pular para campo 49 (hematócrito)
Data da internação	Data		DT_INTERNA		Data de internação, caso tenha ocorrido	
UF de hospitalização	Caracter	2	UF		Sigla da UF onde o paciente foi internado	
Município do hospital	Caracter	7	MUNICIPIO		Nome completo do município onde o paciente foi internado	
Nome do hospital	Caracter	7	HOSPITAL		Nome completo do Hospital onde o paciente foi internado	
Endereço do hospital	Caracter	60	ENDERECO			

Telefone	Caracter	17	TELEFONE			
Data da coleta	Data		DT_COL_HEM		Data da coleta de sangue para exame de hematócrito	
**CAMPO DA FICHA**	**BANCO DE DADOS (DBF)**					
**NOME**	**TIPO**	**TAM**	**NOME**	**CATEGORIAS**	**DESCRIÇÃO**	**CARACTERISTICAS/ CRÍTICA DE CONSISTENCIA**
Dados laboratoriais: hematócrito (maior)	Numérica		HEMA_MAIOR		Resultado do hematócrito (em %, maior valor)	4 dígitos sendo um decimal
Data da coleta	Data		DT_COL_PLQ		Data da coleta sangue para exame de plaquetas	
Plaquetas	Numérica		PALQ_MAIOR		Resultado do exame (plaquetas- em mm3, maior valor)	
Data da coleta	Data		DT_COL_HE2		Data da coleta de sangue para exame de hematócrito	
Hematócrito (menor)	Numérica		HEMA_MENOR		Resultado do hematócrito (em %, menor valor)	
Data da coleta	Data		DT_COL_PL2		Data do resultado do exame de plaquetas	
Plaquetas	Numérica		PLAQ_MENOR		Resultado do exame (plaquetas- em mm3, menor valor)	
Sorologia<br>Data da coleta da 1ª Amostra	Data		DT_SORO1		Data da realização da coleta da 1ª sorologia	Data 1ª COLETA > ou = DT NOTIF.
Sorologia<br>Data da coleta da 2ª Amostra	Data		DT_SORO2		Data da realização da coleta da 2ª sorologia	DT 2ªCOLETA que DT 1ª COLETA
Sorologia<br>Data do resultado da 1ª amostra	Data		DT_SOROR1		Data de recebimento do resultado da 1ª amostra pela VE	DT 1º REC. RESUL. > DT 1ª COLETA
Data do resultado da 2ª amostra	Data		DT_SOROR2		Data de recebimento do resultado da 2ª amostra pela VE	DT 2º REC. RESUL. > DT 2ª COLETA
Resultado exame<br>IgM S1	Caracter	1	S1_IGM	1- reagente<br>2- não reagente<br>3- inconclusivo<br>4- não realizado	Resultado da 1ª sorologia - IgM	
Resultado exame<br>IgM S2	Caracter	1	S2_IGM	1. reagente<br>2. não reagente<br>3. inconclusivo<br>4. não realizado	resultado 2ª sorologia, – IgM	

CAMPO DA FICHA	BANCO DE DADOS (DBF)					
Resultado exame IgG S1	Caracter	1	S1_IGG	1. reagente<br>2. não reagente<br>3. inconclusivo<br>4. não realizado	resultado da 1ª sorologia - IgG	
**CAMPO DA FICHA**	**BANCO DE DADOS (DBF)**					
**NOME**	**TIPO**	**TAM**	**NOME**	**CATEGORIAS**	**DESCRIÇÃO**	**CARACTERISTICAS/ CRÍTICA DE CONSISTENCIA**
Resultado exame IgG S2	Caracter	1	S2_IGG	1. reagente<br>2. não reagente<br>3. inconclusivo<br>4. não realizado	resultado da 2ª sorologia – IgG	
TítulosS1	Número		S1_TIT1	1: _ _ _ _	Resultado dos títulos para a 1ª sorologia	4 dígitos
Títulos S2	Número		S2_TIT1	1: _ _ _ _	Resultado dos títulos para a 1ª sorologia	4 dígitos
Isolamento de vírus – colheu material	Caracter	1	MATERIAL	1- sim<br>2- não<br>9 – ignorado	Se foi colhido material para realização de isolamento viral	Se categoria=2 ou =9, pular para campo 65 (Histopatologia)
Tipo de material Soro na 1ª amostra	Caracter	1	SORO1	1- sim<br>2- não<br>9. ignorado	Se foi colhido soro na 1ª amostra para realização de isolamento viral	
Tipo de material Soro na 2ª amostra	Caracter	1	SORO2	1. sim<br>2. não<br>9. ignorado	Se foi colhido soro na 2ª amostra para realização de isolamento viral	
Tipo de material Tecido	Caracter	1	TECIDOS	1. sim<br>2. não<br>9. ignorado	Se foi colhido tecido para realização de isolamento viral	
Resultado	Caracter	1	RESUL_VIRA	1- Sorotipo I<br>2- Sorotipo II<br>3- Sorotipo III<br>4-Sorotipo IV<br>5-Não detectado	Sorotipo	
Patologia Histopatologia	Caracter	1	HISTOPA	1. compatível<br>2. negativo<br>9. não realizado		
Imunohistoquímica	Caracter	1	IMUNOH	1. positivo<br>2. negativo<br>3. não realizado		

PCR<br>Tipo de amostra	Caracter	1	AMOS_PCR	1. soro<br>2. tecidos		
Resultado	Caracter	1	RESUL_PCR	1. positivo<br>2. negativo		
Outro tipo de amostra	Caracter	1	AMOS_OUT	1- soro<br>2- tecidos		
**CAMPO DA FICHA**	**BANCO DE DADOS (DBF)**					
**NOME**	**TIPO**	**TAM**	**NOME**	**CATEGORIAS**	**DESCRIÇÃO**	**CARACTERISTICAS/<br>CRÍTICA DE CONSISTENCIA**
Técnica Utilizada	Caracter	20	TECNICA			
Resultado	Caracter	1	RESULT_OUT	1- positivo<br>2- negativo		
Classificação final	Caracter	1	COM_CLASSI	1- Dengue Clássico<br>2- Dengue com complicações<br>3- Febre Hemorrágica do Dengue – FHD<br>4- Síndrome de choque da Dengue<br>5- Descartado	Conclusão da investigação	
Critério de confirmação/ descarte<br>No caso de FHD	Caracter<br><br>Caracter	1<br><br>1	CON_CRITER<br><br>CON_FHD	1. Laboratorial<br>2. Vínculo Epidemiológico<br>1. Grau I<br>2. Grau II<br>3. Grau III<br>4. Grau IV	Critério de confirmação/ descarte	Habilitar apenas para a categoria 3 (FHD) no campo classificação final
Local provável da fonte de infecção<br>bairro	Caracter	9	CON_INF_BA		Identificar o bairro da provável da fonte de infecção	
Local provável da fonte de infecção<br>Distrito	Caracter	4	CON_INF_DI		Identificar o distrito da provável da fonte de infecção	
Local provável da fonte de infecção<br>Município	Caracter	7	CON_INF_MU		Identificar o município da provável da fonte de infecção	
Local provável da fonte de infecção<br>UF	Caracter	2	CON_INF_UF		Identificar a UF provável da fonte de infecção	
Local provável da fonte de infecção<br>País	Caracter	3	CON_INF_PA		Preencher se o caso foi infectado em outro país	
Doença relacionada ao trabalho	Caracter	1	CON_DOENCA		Se o paciente adquiriu a doença em decorrência das condições/situação do trabalho	

Evolução do caso	Caracter	1	CON_ EVOLUC	1. cura<br>2. óbito<br>9- ignorado	evolução do caso	Se categoria = 1 ou 9 pular para dt de encerramento (82)
Data do óbito	Data		CON_DT_OBI		Data do óbito	
Data do encerramento	Data		CON_DT_ENC		Data do encerramento do caso	

REPÚBLICA FEDERATIVA DO BRASIL
MINISTÉRIO DA SAÚDE
ESTADO DE SÃO PAULO
SECRETARIA DE ESTADO DA SAÚDE

SINAN
SISTEMA DE INFORMAÇÃO DE AGRAVOS DE NOTIFICAÇÃO
FICHA DE INVESTIGAÇÃO
DENGUE

Nº

CASO SUSPEITO: Paciente com **febre** com duração máxima de 7 dias, acompanhada de pelo menos **dois dos seguintes sintomas** : cefaléia, dor retroorbital, mialgia, artralgia, prostração, exantema e com exposição à área com transmissão de dengue ou com presença de Aedes aegypti nos últimos quinze dias.

Dados Gerais

1 Tipo de Notificação — 2 - Individual

2 Agravo/doença — **DENGUE** — Código (CID10) **A 90**

3 Data da Notificação

4 UF

5 Município de Notificação — Código (IBGE)

6 Unidade de Saúde (ou outra fonte notificadora) — Código

7 Data dos Primeiros Sintomas

Notificação Individual

8 Nome do Paciente

9 Data de Nascimento

10 (ou) Idade — 1 - Hora 2 - Dia 3 - Mês 4 - Ano

11 Sexo M - Masculino F - Feminino I - Ignorado

12 Gestante 1-1ºTrimestre 2-2ºTrimestre 3-3ºTrimestre 4- Idade gestacional Ignorada 5-Não 6- Não se aplica 9-Ignorado

13 Raça/Cor 1-Branca 2-Preta 3-Amarela 4-Parda 5-Indígena 9- Ignorado

14 Escolaridade
0-Analfabeto 1-1ª a 4ª série incompleta do EF (antigo primário ou 1º grau) 2-4ª série completa do EF (antigo primário ou 1º grau)
3-5ª à 8ª série incompleta do EF (antigo ginásio ou 1º grau) 4-Ensino fundamental completo (antigo ginásio ou 1º grau) 5-Ensino médio incompleto (antigo colegial ou 2º grau)
6-Ensino médio completo (antigo colegial ou 2º grau) 7-Educação superior incompleta 8-Educação superior completa 9-Ignorado 10- Não se aplica

15 Número do Cartão SUS

16 Nome da mãe

Dados de Residência

17 UF

18 Município de Residência — Código (IBGE)

19 Distrito

20 Bairro

21 Logradouro (rua, avenida,...) — Código

22 Número

23 Complemento (apto., casa, ...)

24 Geo campo 1

25 Geo campo 2

26 Ponto de Referência

27 CEP

28 (DDD) Telefone

29 Zona 1 - Urbana 2 - Rural 3 - Periurbana 9 - Ignorado

30 País (se residente fora do Brasil)

Dados laboratoriais e conclusão (dengue clássico)

Inv.

31 Data da Investigação

32 Ocupação

Dados laboratoriais

Exame Sorológico (IgM)

33 Data da Coleta

34 Resultado 1 - Reagente 2 - Não Reagente 3 - Inconclusivo 4 - Não Realizado

Exame NS1

35 Data da Coleta

36 Resultado 1- Positivo 2- Negativo 3- Inconclusivo 4 - Não realizado

Isolamento Viral

37 Data da Coleta

38 Resultado 1- Positivo 2- Negativo 3- Inconclusivo 4 - Não realizado

RT-PCR

39 Data da Coleta

40 Resultado 1 - Positivo 2 - Negativo 3 - Inconclusivo 4 - Não Realizado

Sorotipo

41 1 - DEN 1 2 - DEN 2 3 - DEN 3 4 - DEN 4

Histopatologia

42 Resultado 1 - Positivo 2 - Negativo 3 - Inconclusivo 4 - Não Realizado

Imunohistoquímica

43 Resultado 1 - Positivo 2 - Negativo 3 - Inconclusivo 4 - Não Realizado

Conclusão

44 Classificação
1 - Dengue Clássico
2 - Dengue com Complicações
3 - Febre Hemorrágica do Dengue - FHD
4 - Síndrome do Choque da Dengue - SCD
5- Descartado

45 Critério de Confirmação/Descarte
1 - Laboratório
2 - Clínico-Epidemiológico
3 - Em Investigação

Os casos de dengue com complicações, FHD e SCD: preencher a página seguinte.

Local Provável de Infecção (no período de 15 dias)

46 O caso é autóctone do município de residência? 1-Sim 2-Não 3-Indeterminado

47 UF

48 País

49 Município — Código (IBGE)

50 Distrito

51 Bairro

52 Doença Relacionada ao Trabalho 1 - Sim 2 - Não 9 - Ignorado

53 Evolução do Caso 1-Cura 2- Óbito por dengue 3- Óbito por outras causas 4 - Óbito em investigação 9- Ignorado

54 Data do Óbito

55 Data do Encerramento

Dados clínicos (dengue com complicações, FHD e SCD)

D dos Clínicos - dengue com complicações e FHD

A FHD em geral desenvolve-se entre o 3º e o 5º dia de doença, quando há o recrudescimento da febre. A presença de dor abdominal intensa, hepatomegalia dolorosa, hipotermia com sudorese, letargia/agitação, cianose, arritmias, hipotensão arterial/postural, vômitos persistentes, manifestações neurológicas são indicadores de que o paciente pode evoluir para FHD ou para um quadro mais grave de dengue.

56 Manifestações Hemorrágicas?
1- Sim 2- Não 9- Ignorado

57 Se sim, quais? 1- Sim 2- Não 9- Ignorado
Epistaxe
Gengivorragia
Metrorragia
Petéquias
Hematúria
Sangramento Gastrointestinal
Prova do Laço Positiva

58 Houve extravasamento plasmático?
1-Sim 2-Não 9-Ignorado

59 Se sim, Evidenciado por:
1-Hemoconcentração 2-Derrames cavitários 3-Hipoproteinemia

60 Plaquetas (menor)
mm 3

61 No Caso de FHD/SCD Especificar
1 - Grau I 2 - Grau II 3 - Grau III 4 - Grau IV

62 No Caso de Dengue com complicações, que tipo de complicações?
1-Alterações neurológicas 2-Disfunção cardiorrespiratória 3-Insuficiência hepática 4-Plaquetas <20.000 mm3
5-Hemorragia digestiva 6-Derrames cavitários 7-Leucometria < 1000 8-Não se enquadra nos critérios de FHD

63 Ocorreu Hospitalização?
1 - Sim 2 - Não 9 - Ignorado

64 Data da Internação

65 UF

66 Município do Hospital
Código (IBGE)

67 Nome do Hospital
Código

68 (DDD) Telefone

Informações complementares e observações

Observações Adicionais

Investigador

Município/Unidade de Saúde
Cód. da Unid. de Saúde

Nome
Função
Assinatura
